# Therapeutics, Etc.

**Published:** 1890-12

**Authors:** B. F. Church

**Affiliations:** Austin


### Items of Interest.

[CULLED BY DR. B. F. CHURCH, AUSTIN.]

**Cannabis Indica in Gastric Disorders.**—After a full discussion of the forms of indigestion that are recognized, and the use of cannabis indica in their treatment, Dr. See arrives at the following conclusions:

1. The most convenient form in which to employ the drug is the extract in doses of about three-quarters of a grain a day, divided in three portions. Above this dose the drug is apt to produce unpleasant effects.

2. The drug was chiefly tried on non-organic affections of the stomach. These were divided into two groups. The first included cases in which the gastric juice was altered in composition, especially if there was an excess of hydrochloric acid. The second group consisted only of gastro-intestinal neuroses, in which there was no choice in the digested juices.

3. All these affections—dyspepsias and neuroses—were characterised by five sets of symptoms occurring in various proportion. (a) Pain, local or radiating, arising spontaneously or after food. The variations in appetite belong to this group. (b) Atony of the stomach, with or without dilitation, is almost always present. Vomiting is more frequent in the neurotic cases. (c) Flatulence and eructation in most cases, in the neuroses the gas consists of air which has been swallowed, gases formed by decomposition arise from lactic or acetic acid fermentation, and not from excess of hydrochloric acid. These gases are the cause of the painful symptom known as "heartburn." (d) The gastric digestion of flesh food and hydrochloric acid is in excess, but is deficient when too much lactic or acetic acid is present, and completely in abeyance when there is a deficiency of acid. In neurotic cases gastric digestion is normal. Constipation is the rule in most cases. (e) In this last group are placed the varied symptoms, giddiness, migraine, palpitation, agoraphobia, etc.

4. Cannabis indica gives relief from pain and increases the appetite in all cases, no matter on what causes the pain and loss of appetite may depend. If, however, too much hydrochloric acid be excreted, it is better to aid the action of the drug by large doses of bicarbonate of soda given about four hours after food. Cannabis indica has no beneficial action on the atonic state of the stomach, but it relieves vomiting and cramp of the stomach. The drug has no direct influence in checking flatulence, but it aids the expulsion of gas and diminishes heartburn. The digestion of food is improved, if the failure depends upon neuro-paralitic conditions, or is rendered painful by an excess of acid, but no improvement is produced if the disorder is caused by a want of acid. As regards the other symptoms, giddiness, sleeplessness, palpitation and the like some relief is generally experienced by the use of this drug, but no alteration for the better is noticed in the hypochondriacal, hysterical or neurasthenic condition. In short, cannabis indica may be said to be a true sedative to the stomach, without causing any of the inconveniences experienced after the administration of opium, chloral or the bromides. It should be combined with the use of alkalies in large doses and with mild aperients.—*The Lancet, N. Y. Record.*

---

Dr. Roberts Bartholow, in an article on the treatment of croupous pneumonia, published in the *American Journal of the Medical Sciences*, Nov., 1890, bases his treatment upon the theory of the disease being specific. His method seems very rational, whether or not the pneumo-coccus is a factor in its etiology.

The inhalation of ethel iodide is given the first place as a therapeutic agent. He says: "The influence exerted by it upon the cough, the labored breathing and general *malaise* is a remarkable fact. It has the signal advantage that no apparatus is required, it vaporizes at ordinary temperature, it causes no irritation of the mucous membrane, and it does not act as an anæsthetic. It can be given on a folded handkerchief, dropped in such doses as may be necessary, or it may be inhaled from a wide-mouthed vial. As full inspiration as the patient can make, is then practiced, and the vapor thoroughly diffused through the air glides into the utlimate air sacs, whence, also, it passes into the blood.

The physiological action of ethel iodide is antiseptic and depurative or eliminant. * * * "The effects of ethel iodide are both local and systemic—locally upon the parts impinged upon in entering the system, and systemic in that, diffusing into the blood, it is necessarily carried to all parts.

What explanation solves may be true of its action, all of the cases were apparently much benefited by the inhalation, and all expressed a sense of relief to the general *malaise*, and to the specific symptom, cough, bronchial irritation, and expectoration, —the last mentioned being soon changed in character and in quantity. The manner of conducting the inhalation consisted in dropping the requisite dose (m. xx to ʒss.) on a folded handkerchief tightly placed over the mouth and nose, by the patient if able to do so, or by the nurse, when necessary. No complaint was made of the action, except some dizziness, and this occurred in a few only."

Small doses of calomel, 3 grs., repeated two or three times, if necessary, are given to relieve portal congestion; acting in this way as an antipyretic, it also has an antiseptic effect.

For sleeplessness and delirium in the early stages he recommends chloral in moderate doses, and agrees with Dr. Ben Richardson, that chloral has the power to dissolve fibrin masses and exudates. He favors blood-letting by leeches in some cases, but thinks venesection is rarely necessary. Food or stimulants should not be forced upon an unwilling stomach. As three or four hours is the minimum time for stomach digestion, food, under no conditions, should be given at shorter intervals. Stimulants only when there is marked depression. If necessary, paregoric may be given for pain and cough. Besides its systematic effects, the iodide will greatly modify the last symptom.

---

Lupus Vulgaris.—Lupus vulgaris, of which we are now hearing so much, is an extremely chronic disease of the skin, attacking persons between the ages of two and fifteen. It is characterized, says Green, by the appearance of reddish-brown nodules of granulation tissue upon the skin, usually of the face. The mucous membranes are rarely affected. The nodules start in the corium, but penetrate the connective tissue beneath and the papillary layer above. The different stages and clinical aspect of the disease as it progresses are known as lupus maculatus, lupus ufoliatinus, lupus exulcerans, lupus serpiginosus, and lupus hypertrophicus.

The disease shows by the formation of fresh nodules at the perifery of the original lesion, new centers form and the old ones may gradually disappear. If the tissue breaks down, an open sore is found, covered with yellowish and brownish crusts.

The nodules consist of granulation tissue, containing epithelioid and a few giant cells. Unlike ordinary tubercle, the lupus nodules are rather vascular. Tubercle bacilli are found in the tissue, but they are very infrequent, and often many examinations are required to detect them. Inoculation of lupus nodules will, it is asserted, cause tuberculosis in rabbits and guinea pigs, but inoculation of the skin with tubercle will not produce lupus.

For this and other reasons so distinguished an authority as Kaposi denies that lupus is a cutaneous tuberculosis, although that view is held positively by Koch and his pupils.

Lupus, chronic as it is in its tendencies, often disappears for a time under treatment, only to reappear later. Dermatologists generally give a favorable prognosis, provided treatment is persisted in.—*N. Y. Med. Record, Nov. 20, '90.*

---

**Local Anæsthesia for Slight Operations.**—For operations upon small abscesses, opening fistulous tracts, or removing superficial growths, it is recommended that local anæsthesia be secured by atomization of the following solution:

℞ Chloroform 10 parts,
Sulphuric ether 15 parts,
Menthol 1 part
M.

The anæsthesia thus obtained lasts two to ten minutes.—*Medical News.*

---

**Aristol in Otorrhœa.**—Dr. Williams, in the St. Louis *Surgical and Medical Journal*, found good results by the use of Aristol, in connection with boric acid, in the treatment of otorrhœa. His treatment was to wash the ear thoroughly and dry it, then apply aristol powder, and follow this with boric acid. Thinks the use of aristol a good preparatory treatment and that a single application facilitates a cure.

